# Understanding the Effect of Energy Density and Formulation Factors on the Printability and Characteristics of SLS Irbesartan Tablets—Application of the Decision Tree Model

**DOI:** 10.3390/pharmaceutics13111969

**Published:** 2021-11-20

**Authors:** Marijana Madžarević, Đorđe Medarević, Stefan Pavlović, Branka Ivković, Jelena Đuriš, Svetlana Ibrić

**Affiliations:** 1 Department of Pharmaceutical Technology and Cosmetology, Faculty of Pharmacy, University of Belgrade, Vojvode Stepe 450, 11221 Belgrade, Serbia; djordje.medarevic@pharmacy.bg.ac.rs (Đ.M.); jelena.djuris@pharmacy.bg.ac.rs (J.Đ.); svetlana.ibric@pharmacy.bg.ac.rs (S.I.); 2 Institute of Chemistry, Technology and Metallurgy, University of Belgrade, Njegoseva 12, 11001 Belgrade, Serbia; stefan.pavlovic@ihtm.bg.ac.rs; 3 Department of Pharmaceutical Chemistry, Faculty of Pharmacy, University of Belgrade, Vojvode Stepe 450, 11221 Belgrade, Serbia; branka.ivkovic@pharmacy.bg.ac.rs

**Keywords:** printability, laser speed, energy density, decision tree, tailoring drug release

## Abstract

Selective laser sintering (SLS) is a rapid prototyping technique for the production of three-dimensional objects through selectively sintering powder-based layer materials. The aim of the study was to investigate the effect of energy density (ED) and formulation factors on the printability and characteristics of SLS irbesartan tablets. The correlation between formulation factors, ED, and printability was obtained using a decision tree model with an accuracy of 80%. FT-IR results revealed that there was no interaction between irbesartan and the applied excipients. DSC results indicated that irbesartan was present in an amorphous form in printed tablets. ED had a significant influence on tablets’ physical, mechanical, and morphological characteristics. Adding lactose monohydrate enabled faster drug release while reducing the possibility for printing with different laser speeds. However, formulations with crospovidone were printable with a wider range of laser speeds. The adjustment of formulation and process parameters enabled the production of SLS tablets with hydroxypropyl methylcellulose with complete release in less than 30 min. The results suggest that a decision tree could be a useful tool for predicting the printability of pharmaceutical formulations. Tailoring the characteristics of SLS irbesartan tablets by ED is possible; however, it needs to be governed by the composition of the whole formulation.

## 1. Introduction

Nowadays, we are faced with the rapid development of three-dimensional (3D) printing techniques in pharmaceuticals. Three-dimensional printing brings unprecedented opportunities to individualize medicine to a patient’s body weight and lifestyle by dose and dosage form adjustment [1]. Various 3D printing technologies have been intensively investigated in the past decade in pharmaceutical fields, such as fused filament fabrication (FFF), stereolithography (SLA), digital light processing (DLP), selective laser sintering (SLS) and 3D inkjet printing [2,3,4].

SLS is a rapid prototyping technique for the production of 3D objects through selectively sintering powder-based layer materials [2,5,6]. During the printing process, the laser is directed to draw a specific pattern onto the surface of the powder bed. Once the first layer is completed, the powder bed moves down to the z-axis, the reservoir moves up and a roller distributes a new layer of powder on top of the previous one [7]. The process is repeated many times until the desired object is created. The method is solvent free and does not require a filament form of raw material or photopolymerizable resin. Additionally, there is no requirement for the addition of liquid binder [7,8]. The main disadvantage of SLS technology is that it requires a high temperature; thus, SLS printing is appropriate for thermostable substances. In order to obtain the dosage form of the desired characteristics, it is necessary to understand the printing process from the choice of material to the characteristics of the final form.

The most commonly used excipients in SLS printing of dosage forms are lactose monohydrate [7,9,10], Kollidon^®^ VA 64 [10,11,12], Kollicoat^®^ IR [5], hydroxypropyl methylcellulose [12], ethyl cellulose [8,13] and Eudragit^®^ L100-55 [5,13]. Particles should have good flow properties and optimum size and shape [14,15]. The recommendation for the optimal SLS printing process is that the particle size should be from 58 to 180 µm [6]. In the literature, there is a limited number of studies in the pharmaceutical field characterizing the properties of powder mixtures in SLS printing. They were mostly based on an investigation of their thermal characteristics. Thakkar et al. characterized powder flow and the angle of response for tested formulations [16].

The development of drug-delivery devices by SLS has been conducted using biodegradable and biocompatible polymers [17,18,19,20,21]. According to a recent published review paper, fourteen publications on SLS applied to pharmaceutical research on solid oral forms were identified in the MEDLINE^®^ database from 2017 to 2020 [15]. The first report on the use of SLS in the preparation of oral dosage forms was in 2017, and it explored the drug release properties of printed tablets [5]. Studies are, then, geared towards the development of orally disintegrating printlets [12,22], drug-loaded gyroid lattices [13], miniprintles [8], printlets with braille and moon patterns [23], amorphous solid dispersion dosage forms [24] and high-dose controlled release pharmaceutical dosage forms [25]. A recent study combined the application of twin-screw granulation and selective laser sintering 3D printing for the development of pharmaceutical dosage forms with enhanced dissolution rates and physical properties [16]. The obtained dosage forms were characterized in terms of solid-state and morphological characteristics, mechanical properties, drug content, disintegration time and drug release testing.

Laser power, bed temperature, and layer thickness are recognized as the most important process parameters which affect the quality of produced objects [26]. In a study by Fina et al., the possibility of modulating drug release properties with different laser speeds was demonstrated [12]. In a study by Barakh Ali et al., the formulation and process variables affecting the quality of the printlets were investigated using the Box–Behnken response surface methodology [10]. Response surface methodology was also used for the optimization of formulations [7]. Energy density (ED) could be considered a critical parameter in SLS printing [15]. ED is the amount of energy transmitted per volume unit [27]. It depends on four process parameters and it could help standardize the interpretation of the results between different printers [15].

Irbesartan, the model drug in this study, is used for the treatment of hypertension and belongs to Class II drugs according to the Biopharmaceutical Classification System (BCS); i.e., it has low solubility and high permeability. Irbesartan was used as one of the five drugs in the production of SLA 3D multi-layer antihypertensive polypill [28] and this is the first time it has been applied in SLS 3D printing.

A recently published study showed that photo-absorber loading and ED could be critical for printing efficiency and dimensional accuracy [29]. Printability, an important aspect for understanding the sintering process has been evaluated in one article, and the need for more detailed research has been recognized [15]. The main purpose of this manuscript was to investigate the effect of ED and formulation factors on printability using a decision tree as a data mining tool, and then to evaluate the effects of these factors on the characteristics of the produced SLS irbesartan tablets.

## 2. Materials and Methods

### 2.1. Materials

Irbesartan, a model active substance, was donated by Hemofarm (Vrsac, Serbia). Lactose monohydrate was purchased from CARLO ERBA (Milan, Italy). Vivapharm^®^ E3, (hydroxypropylmethylcellulose, HPMC) was obtained from JRS Pharma (Rosenberg, Germany). Mannitol Parteck^®^ M 200 and Candurin^®^ Gold Sheen were obtained from Merck, Darmstadt, Germany. Kollidon^®^ VA 64 Fine (vinylpyrrolidone-vinyl acetate copolymer) was supplied from BASF, Ludwigshafen, Germany. Crospovidone NF (Polyplasdone^®^ XL-10) was obtained from Ashland (Wilmington, DE, USA) and AEROSIL^®^ 200 (colloidal silicon dioxide) was provided by Evonik (Essen, Germany).

### 2.2. Methods

#### 2.2.1. Preparation of Powder Blends

Different formulations prepared for 3D printing are shown in Table 1. Candurin^®^ Gold Sheen was added to enhance energy absorption from the laser and to aid printability in the SLS process [12]. Formulations FM1 and FM2 contained mannitol, whereas formulations FH1–FH8 contained HPMC as the excipient, which is present in the largest percentage. In formulations FH2, FH3, FH4 and FH8, crospovidone, as a super disintegrant, was added to increase the disintegration time which would allow for a faster drug release. FH5 contained Kollidone^®^ VA 64 Fine, whereas FH6, FH7 and FH8 contained lactose monohydrate to investigate their effects on the printability and characteristics of tablets. For all the formulations, 150 g of a mixture containing a drug and excipients were blended in a powder mixer (Farmalabor, Canosa di Puglia, Italy) for 9 min at 60 rpm. Afterward, colloidal silicon dioxide (1% *w*/*w*) was added, and the powder was mixed for an additional one minute.

#### 2.2.2. Characterization of Powder Blends

##### Particle Size Distribution

Particle size distribution was analyzed by sieving using a vibrating shaker (ERWEKA AR400, Heusenstamm, Germany) and six standard sieves in the range of 45–500 µm. The sample mass was 100.00 g. The amount of material that remained on each sieve was accurately weighted to determine the particle size distribution of HPMC, mannitol, lactose monohydrate and each formulation.

##### Powder Flow Properties

The flowability of powder was measured by the indirect method, determining the bulk and tapped densities according to the Ph. Eur. 10.0 recommendations, and by calculating the Hausner ratio and compressibility index (CI). The volumeter STAV 2003 (J. Engelsmann AG, Ludwigshafen, Germany) was used to determine the tapped density. The Hausner ratio and CI were calculated using Equations (1) and (2), respectively, as below (Ph. Eur. 10.0):

(1) $$Hausner\;ratio=\frac{tapped\;density}{bulk\;density}$$

(2) $$Compressibility\;index\;\left(CI\right)=\frac{\left(tapped\;density-bulk\;density\right)}{tapped\;density}\times 100\;$$

The flowability characteristics of the powders was defined following descriptive terms given in Ph. Eur. 10.00.

#### 2.2.3. Preparation of Tablets

##### Selective Laser Sintering 3D Printing

A 3D model of the printed dosage forms (a cylinder 6 mm in diameter, and 3 mm in height) was designed with Autodesk Fusion 360 software version 2.0.8809 (Autodesk Inc., San Rafael, CA, USA) and exported as a stereolithography file (.stl) into the 3D printer software (Sintratec Central, version 1.2.0, Sintratec Kit, AG, Brugg, Switzerland). The blend was filled in the modified powder reservoir compartment of the SLS printer (Sintratec Kit, AG, Brugg, Switzerland) equipped with a 2.3 W 455 nm laser. A powder batch of 100 g was used for each build cycle. The printing parameters were controlled using Sintratec 3D printer software. For each formulation, the printing was tested under different conditions through varying printing temperatures, laser speeds and layer heights, after which the parameters of successful printing were selected (Table 2). Hatch spacing was set at 250 µm. The chamber temperature was kept below the surface temperature for ensuring the proper transfer of the physical mixture to the print chamber [24]. The obtained SLS tablets from the same formulation but with different laser speeds, were marked with P1, P2, etc. Energy density (ED) is the amount of energy transmitted per volume unit. It depends on four process parameters and could be considered a critical parameter in SLS printing [15]. ED was calculated by Equation (3).

(3) $$ED=\frac{LP}{SS\times LS\times HS}$$

where *LP*, *SS*, *HS* and *LT* are, respectively, laser power, scanning speed, hatch spacing and layer thickness. The laser power in the printer used in this study, the Sintratec Kit, is not tunable. Hatch spacing was kept constant. Layer height was adjusted to the particle size. The evaluation of *ED* as critical process parameters in the SLS printing of solid dosage forms could allow for the comparison of the results between different printers. Hence, in this work, *ED* was studied through a change in laser speed.

#### 2.2.4. Decision Tree Modelling

Applying a decision tree has been proved to be an effective and reliable technique [30]. It mimics human decision making and it can be easily visualized in the form of a tree, making it easy to convey and interpret results [31]. In order to better understand the printability of prepared HPMC physical mixtures, RapidMiner Studio software version 9.9 (RapidMiner Inc., Boston, MA, USA) was used. The input data used to generate the decision tree were the following: ED, particle size distribution, the content of crospovidone, HPMC, Kollidon^®^ VA 64 Fine and lactose monohydrate. Printability (categorized as yes and no) was set as a target role. The data were split between training (70%) and test dataset (30%). The minimal leaf size varied from 1 to 5, a minimal gain from 0.01 to 0.05. A maximal tree depth from 5 to 10 was used in the study in order to obtain a decision tree with the best accuracy. The optimal combination of parameters is determined by trying out all possibilities. Accuracy was calculated as a percentage of correct predictions. A pruning technique (pre-pruning and post-pruning) was used for preventing the overfitting of the data. A confidence level of 0.1 was selected for the pessimistic error calculation of pruning.

#### 2.2.5. Characterization of the Tablets

##### Determination of the Physical and Mechanical Properties

Tablets (*n* = 10) were weighed on a Sartorius BP 210 D analytical balance (Sartorius, Goettingen, Germany) and measured (diameter and thickness) using a digital caliper (Vogel, Kevelaer, Germany). The breaking force of the tablets (*n* = 10) was measured using the ERWEKA TBH 125D (ERWEKA, Langen, Germany), a hardness tester.

##### Mercury Intrusion Porosimetry (MIP) Measurements of Irbesartan Tablets

MIP measurements were performed in a fully automated conventional apparatus, the CARLO ERBA Porosimeter 2000 (Carlo Erba, Milan, Italy), pressure range: 0.1–200 MPa; pores with a diameter between 7.5 and 15,000 nm. The acquisition of the analysis data was performed using the Milestone Software 200 (Carlo Erba, Milan, Italy). Two subsequent intrusion–extrusion runs (Run I and Run II) were conducted. The samples were evacuated for 2 h in a dilatometer placed in the Macropores Unit 120.

##### Scanning Electron Microscopy (SEM)

The morphology of the tablets’ cross-sections was investigated using SEM. Samples were coated with gold alloy at 30 mA for 100 s on the BAL-TEC SCD 005 sputter coater (Leica Microsystems, Wetzlar, Germany) to improve their conductivity during recording. Micrographs were taken with the JEOL JSM-6390LV scanning electron microscope (JEOL, Tokyo, Japan) at suitable magnifications.

##### Fourier Transform Infrared Spectroscopy (FT-IR)

FT-IR spectra of raw materials and crushed tablets were recorded using the Nicolet iS10 (Thermo Scientific, Waltham, MA, USA) FT-IR spectrometer, equipped with a single reflection ATR system (Smart iTR, Thermo Scientific, Waltham, MA, USA) with a diamond plate and ZnSe lens. The spectra were collected as an average of 16 scans in the frequency range from 4000 to 650 cm^−1^, with a resolution of 2 cm^−1^.

##### Differential Scanning Calorimetry (DSC)

In this study, DSC was used to investigate the physical state of the drug in the formulations. DSC analyses were performed on a Mettler Toledo AG DSC 1 differential scanning calorimeter (Analytical, Zurich, Switzerland). Accurately weighed 5–10 mg samples were placed in pierced aluminum pans and subjected to heating at 10 °C/min in the range from 25–250 °C under a nitrogen purge gas flow of 50 mL/min. An empty pan was used as a reference.

#### 2.2.6. Disintegration

The tablets’ disintegration time was measured according to the Ph. Eur. 10.0 procedure, in a compendial ERWEKA ZT 52 disintegration tester (Erweka GmbH, Langen, Germany), using 800 mL of purified water as media at 37 ± 0.5 °C. One tablet was placed in each of the six tubes of the basket, and covered with a disc.

#### 2.2.7. Drug Content

Tablets (*n* = 3) were crushed using a mortar and pestle. Approximately 100 mg was diluted with 10 mL methanol. Samples were placed in a Bandelin Sonorex RK102H ultrasonic bath (Bandelin Sonorex, Berlin, Germany) at room temperature and sonicated for 15 min to enhance the extraction of irbesartan. After filtration, 1 mL of the solution was diluted with 0.1 M HCl. The amount of drug in the solution was determined UV spectrophotometrically using the Evolution 300 (Thermo Fisher Scientific, Waltham, MA, USA) at 225 nm. Because no excipients are absorbed at the same wavelength as irbesartan, UV spectrophotometry was used as the method of choice. The measurement was done in triplicate. The obtained drug content was expressed as a percentage of the theoretical drug content ± standard deviation (S.D.).

#### 2.2.8. Dissolution and Drug Release Analysis

Drug dissolution profiles for the 3D tablets were obtained with the paddle apparatus ERWEKA DT 600 (Erweka GmbH, Langen, Germany) in 500 mL of 0.1 M HCl at 37 ± 0.5 °C with a paddle speed of 50 rpm. Samples were withdrawn at a predetermined time interval and the absorbance of the dissolved irbesartan was measured UV spectrophotometrically at the wavelength of the relative maximum absorbance of irbesartan (225 nm). The analysis was performed in triplicate. FH8 P1, P2 and P3 tablets exhibited floating characteristics and they were investigated in the basket apparatus in 500 mL 0.1 M HCl at 37 ± 0.5 °C, with the rotation speed of 100 rpm. This speed is discriminatory for the basket method [32].

Therefore, drug release profiles were fitted into four mathematical models including zero-order, first-order, Higuchi, and Korsmeyer–Peppas.

## 3. Results

### 3.1. Characterization of Powder Blends

**Figure 1:**
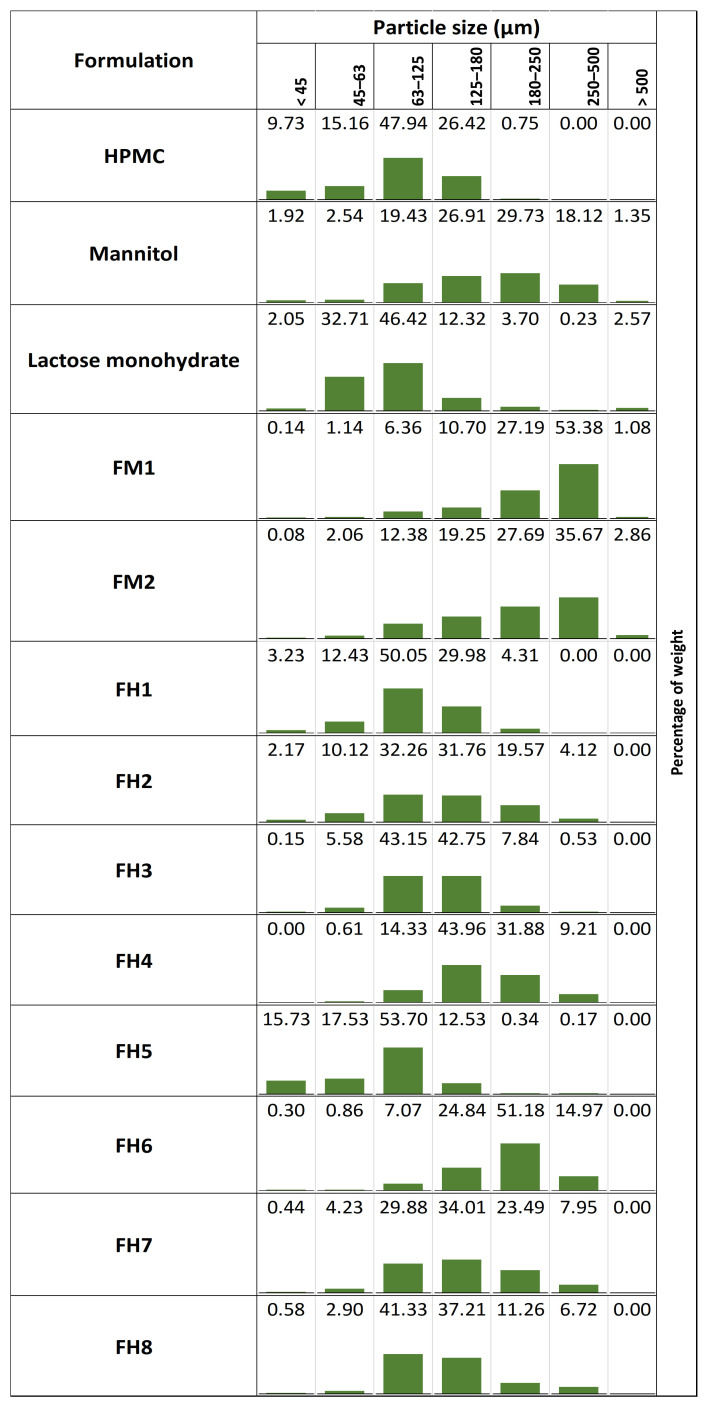
Particle size distribution of powder mixtures for 3D printing.

The particle size from 58 to 180 µm and good flowability characteristics are described as optimal for the SLS printing process [6]. Guided by this, powders with poor flow characteristics were excluded from the study and the data are not shown. Mannitol (Parteck^®^ M 200) was chosen because of its unique surface structure, and HPMC was chosen because of its printable properties which have previously been described in the literature. Additionally, mannitol and HPMC have melting temperatures (165 °C and 160 °C, respectively) that are within the operating range of the SLS printer. The particle size distribution is shown in Figure 1. It can be observed that in formulations FM1 and FM2, the most common are particles 250–500 µm in size. In other formulations, the most common are particles 65–250 µm in size. These results are in accordance with the particle size distribution of mannitol and HPMC.

From the results in Table 3, it can be concluded that formulation FM1 had fair flow characteristics, whereas FM2 had good flow characteristics. They had better flowability than other formulations due to the presence of mannitol obtained in the spray granulation process. All other formulations had passable flow characteristics, except FH8, which had poor flow characteristics. According to the results, FM1 and FM2 were not printable due to having a particle size larger than 250 µm, despite satisfactory flowability. The choice of material and the definition of its desirable characteristics have been recognized as the greatest challenges in SLS printing [7]. Insufficient information on the use of pharmaceutical excipients in SLS printing creates a need for detailed investigation. In this study, HPMC E3 proved to be an excellent excipient for SLS printing. However, for better understanding the printability of HPMC physical mixtures, a data mining tool was used and is described further in the text.

### 3.2. Preparation of Tablets

**Figure 2:**
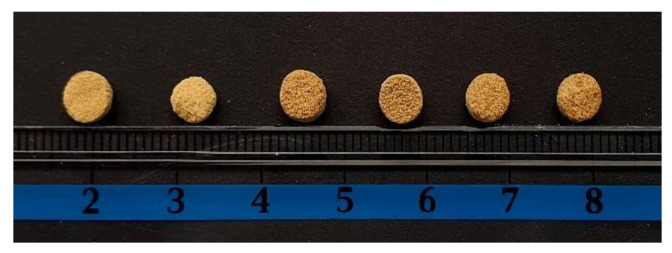
Photographs of the selected SLS-printed tablets with irbesartan. Tablets were printed with different laser speeds. From left to right: 100.00 mm/s, 120.00 mm/s, 140.00 mm/s, 160.00 mm/s, 180.00 mm/s, and 220.00 mm/s.

The biggest challenge with the SLS printing was finding appropriate printing parameters. In most studies, there are only reports on the temperature used without any details on how the temperature was determined. The literature suggests that the powder bed temperature should be set at least 3–4 °C lower than their melting point or close to their glass transition temperature [6]. Moreover, the chamber temperature should be set below the surface temperature [24]. Printing parameters were investigated with a trial and error approach. Parameters were considered unsuitable when the obtained tablets became darker and burned on the surface. Further attempts were made with lower temperatures and higher laser speeds. If tablets disintegrated in the hand upon removal from the chamber, the printing parameters were also considered unsuitable. After selecting the successful printing parameters, tablets were printed with an SLS printer (Figure 2). During one print, it was possible to print up to ten tablets, and the time required for printing one set of tablets was 17 min and 51 s. All tablets in one print were successfully printed.

### 3.3. Decision Tree

**Figure 3:**
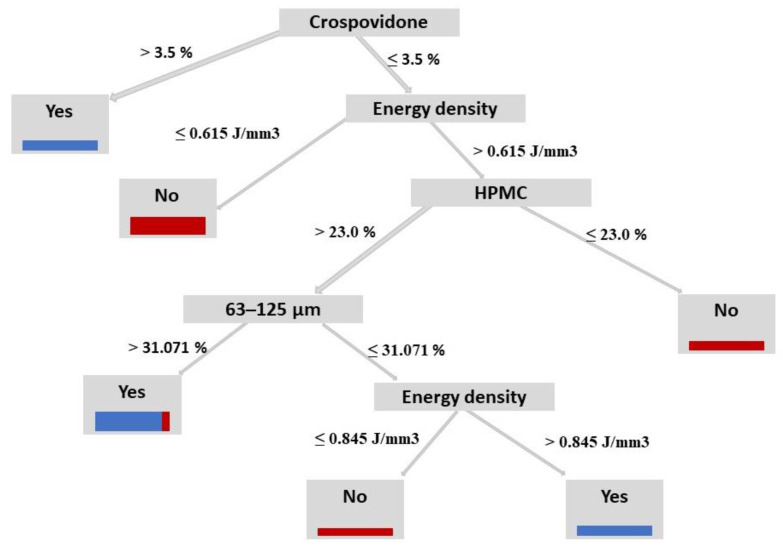
Decision tree for SLS printability of powder mixtures with irbesartan.

The printability of pharmaceutical materials is influenced by formulation characteristics as well as process conditions [15]. The printability of raw materials, including drugs and excipients, was evaluated in a study conducted by Yang et al. [29]. The authors evaluated photo-absorber loading and energy density (ED) on printing efficiency and dimensional accuracy. In this study, decision tree modeling was used to understand the printability factors of physical mixtures with HPMC for SLS printing. A decision tree was developed by testing several values for minimal leaf size, minimal gain and maximal tree depth and selecting the tree with the best accuracy. The decision tree model ran with a minimal leaf size of 2 and a minimal gain of 0.01 to produce a split, and a maximal tree depth of 10 achieved the best overall accuracy of 80.00%. A created decision tree is provided in Figure 3. It demonstrated how printability is affected by both formulation and ED. The most important factor that affected printability was the content of crospovidone. If the content of crospovidone is greater than 3.5%, the formulation is printable. If the content is less than 3.5%, then it is important to consider the ED. If the ED is less than 0.615 J/mm^3^, then the formulation is not printable. Otherwise, it is necessary to consider the content of HPMC. Results suggest that a decision tree could be a useful tool in understanding the SLS printability of pharmaceutical materials. Decision trees are powerful classification algorithms that are used in medicine and other areas [30,33,34,35]. Decision trees have been also used to analyze the printability of filaments in the FDM technique [36] but there is no literature data on the application of decision tree methodology in SLS printing. Created decision trees can be used for predicting printability instead of usage in a trial-and-error approach, which is both time- and resource-consuming. Similar to most machine learning algorithms, the employed methodology is data-dependent. Further investigation with larger data sets could be a step forward in the optimization of formulation and process development of SLS printing.

### 3.4. Effect of Formulation Factors on Printability

In a study by Allahham et al., Mannitol Parteck^®^ Delta M was used as a filler for the production of SLS orally disintegrated tablets [22]. In this study, Mannitol Parteck^®^ M 200 was used, although formulations FM1 and FM2 could not be considered printable because the tablets achieved very poor mechanical characteristics and they disintegrated in the hand upon removal from the chamber. The next choice was HPMC due to its melting temperature (160 °C) that is within the operating range of the SLS printer. HPMC has shown appropriate characteristics for SLS printing, and all FH formulations were printable. For the first time, crospovidone was used for SLS printing. It was added as a super disintegrant, but due to the melting temperature (150 °C) which is close to the printing temperature, a more efficient sintering process occurred and sintering was enabled at different laser speeds. Formulation FH4 (crospovidone 5%) was printable with a wide range of laser speeds (laser speeds from 100.00 to 220.00 mm/s), hence it was possible to evaluate the effects of laser speed on tablet characteristics. The FH6 (lactose monohydrate 45%) formulation was printable only with 100.00 mm/s; with laser speeds of 120.00 mm/s and faster, the tablets had very poor mechanical characteristics and they were excluded from the study. Formulation FH7 (lactose monohydrate 20%) was printable with a laser speed of 100.00 to 120.00 mm/s. It was reported that the SLS tablets containing a high lactose monohydrate concentration required high energy (slow laser scanning speed and high chamber temperature) to sinter/melt the powder [10]. In addition, lactose monohydrate has a higher melting temperature than polymer. Hence, it can be concluded that the energy transmitted to FH6 and FH7 formulations with a higher laser speed was not sufficient. The formulation FH8 (lactose monohydrate 20%) contained 2% crospovidone, in addition to lactose monohydrate and was printable with laser speeds up to 140.00 mm/s.

### 3.5. Characterization of the Tablets

#### 3.5.1. Physical and Mechanical Properties of Irbesartan Tablets

**Figure 4:**
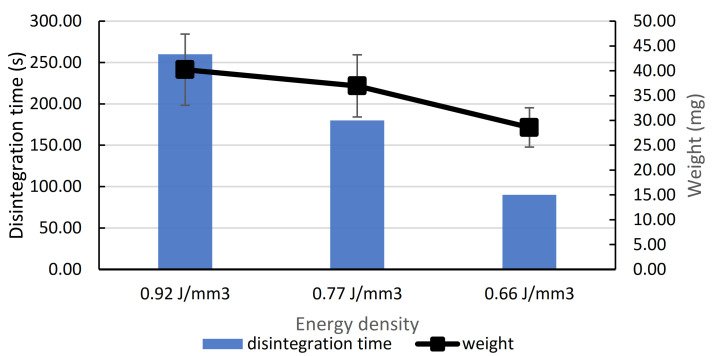
Effect of energy density on the disintegration time and weight of FH8 SLS tablets.

The physical and mechanical properties of irbesartan tablets are summarized in Table 3. It was observed that tablets printed with a slower laser speed tend to be heavier (Figure 4) because powder particles are subjected to higher ED, empty spaces are reduced and there is more room for powder particles to be sintered [12]. This effect of ED on the weight of the tablets could be observed in tablets from formulations with Kolidon VA64 (FH5) and lactose monohydrate (FH7 and FH8; one type of tablet was printed from FH6). For most of the SLS tablets, the breaking force was too low to be detected. This is particularly noticeable in tablets with lactose monohydrate. The negative effect of lactose monohydrate on hardness is reported in the literature [10].

#### 3.5.2. MIP Measurements of Irbesartan Tablets

**Figure 5:**
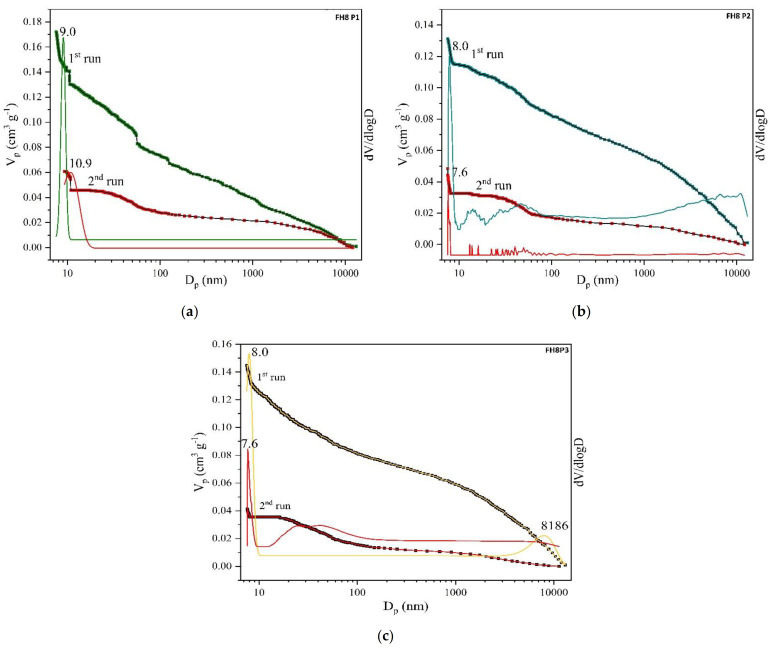
Pore size distribution curves of SLS tablets (**a**) FH8 P1, (**b**) FH8 P2, and (**c**) FH8P3.

MIP measurements of FH8 P1, P2 and P3 irbesartan tablets (*n* = 3) are given in Table 4 and pore size distribution (PSD) is shown in Figure 5. During the first Hg intrusion cycle, mercury filled up both the interparticle (voids) and intraparticle spaces, whereas in the second cycle of the measurement, Hg could occupy only the space of the accessible pores (intraparticle space) [37]. The porosity for FH8 P1, P2 and P3 in the second run was decreased by >60%. That reduction indicates the presence of a specific pore shape, as well as interparticle porosity. Analyzed samples were found to belong to the group of highly macroporous materials. These results are confirmed by SEM micrographs, on which a macroporous structure can be seen. In FH8 P3 tablets, the porosity was about 28.5% of the total value obtained in the first run, leading to the conclusion that more voids between particles were present in FH8 P3 than in FH8 P1 and P2.

**Table 4:** MIP measurements of irbesartan tablets.

	Run	^1^ V_tot_ (cm^3^ g^−1^)	^2^ S_Hg_ (m^2^ g^−1^)	^3^ D_av_ (μm)	^4^ BD (g cm^−3^)	^5^ P (%)
**FH8 P1**	I	0.17	28.00	8.00	1.07	18.40
	II	0.06	7.60	10.00	1.07	6.50
**FH8 P2**	I	0.13	13.40	8.00	1.14	15.00
	II	0.05	10.10	8.00	1.14	5.50
**FH8 P3**	I	0.15	17.80	0.01	1.14	16.50
	II	0.04	5.60	0.01	1.14	4.70

^1^ Total pore volume, ^2^ Specific surface area, ^3^ Pore diameter average, ^4^ Bulk density, ^5^ Porosity.

#### 3.5.3. SEM

**Figure 6:**
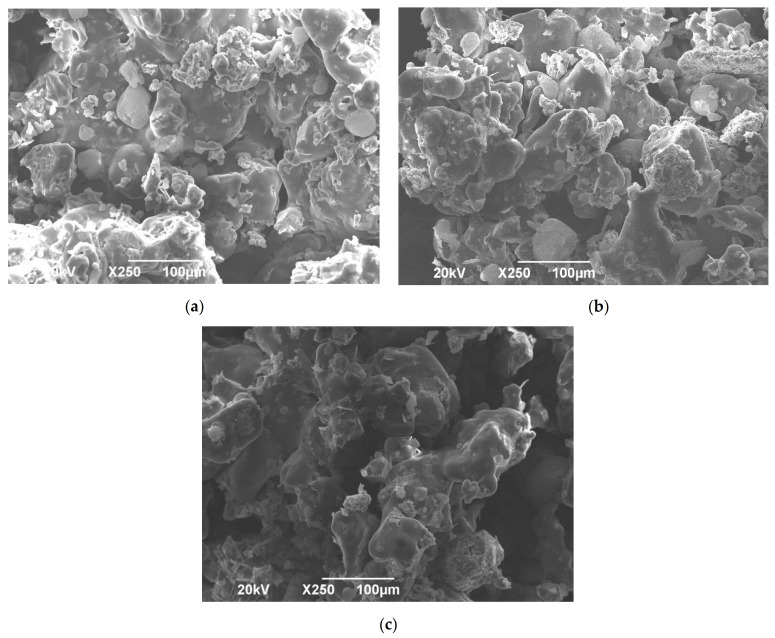
SEM micrographs of the cross-sectional view of irbesartan tablets (**a**) FH8 P1, (**b**) FH8 P2, and (**c**) FH8 P3.

The SEM images provided the morphological properties of the tablets (Figure 6). It can be seen on the SEM micrographs of SLS tablets that some parts are sintered, whereas substance remained in the powder form (Figure 6a–c). These structures create tablets with interparticle pores, although liquid can penetrate quickly, resulting in the rapid disintegration of the tablets. Increasing the laser speed and reducing the ED results in the reduction of the powder particles’ sintering and, consequently, the formation of more interparticle pores (voids) occurs [12]. A faster laser speed (140.00 mm/s) for FH8 P3 tablets led to an increase in voids between particles when compared with FH8 P2 and P1, which printed at 120.00 mm/s and 100.00 mm/s, respectively.

#### 3.5.4. FT-IR

**Figure 7:**
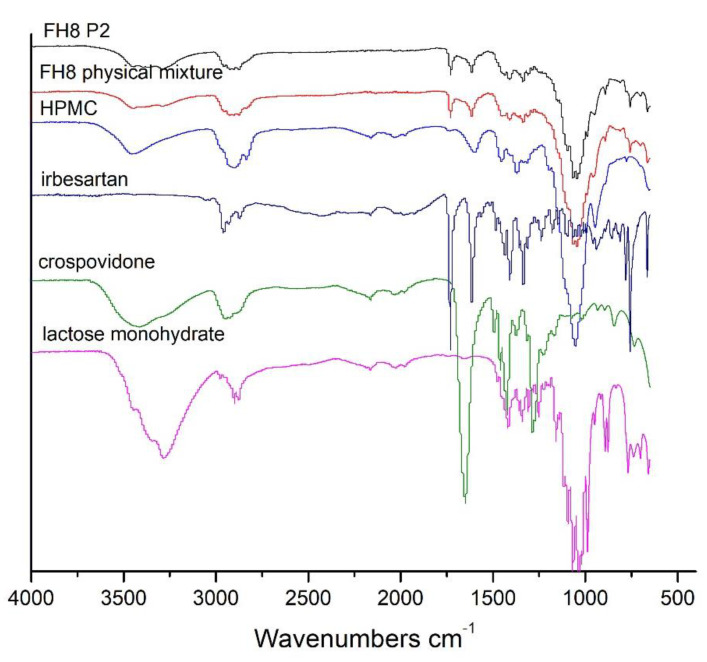
FT-IR spectra of raw materials and evaluated tablet formulations.

Crystalline irbesartan shows characteristic absorption bands at 1730 and 1620 cm^−1^, assigned to the carbonyl moiety of the carboxyl group and the C=N bond in the diazaspiro ring vibrations, respectively (Figure 7). These values are similar to those reported by Skotnicki et al. (1733 and 1617 cm^−1^) and Yan et al. (1732 and 1616) [38,39]. The FT-IR spectrum of HPMC displays a peak at 3450 cm^−1^, revealing the presence of hydroxyl group (OH) stretching. The bend at around 2960 cm^−1^ is attributed to the single bond C-H stretching [40] and the intense peak at 1060 cm^−1^, and represents out-of-phase vibrations associated with an alkyl-substituted cyclic ring containing ether linkages [41,42]. The lactose monohydrate band corresponding to the hydroxyl group (O-H) is a broad peak at 3280 cm^−1^. It also exhibited the characteristic doublet peaks at 1070 and 1030 cm^−1^, corresponding to skeletal vibrations of C-C stretching. These results are verified in a study conducted by Barakh Ali et al. (2019). Crospovidone shows a sharp peak at 1270 cm^−1^ due to C-N stretching, following a literature study performed by Jana et al. (2014) [43]. In the case of the FH8 physical mixture and FH8 P2 characteristics, peaks of irbesartan were observed. Therefore, the FT-IR analyses indicated the absence of any significant interaction between the irbesartan and the excipients used.

#### 3.5.5. DSC

**Figure 8:**
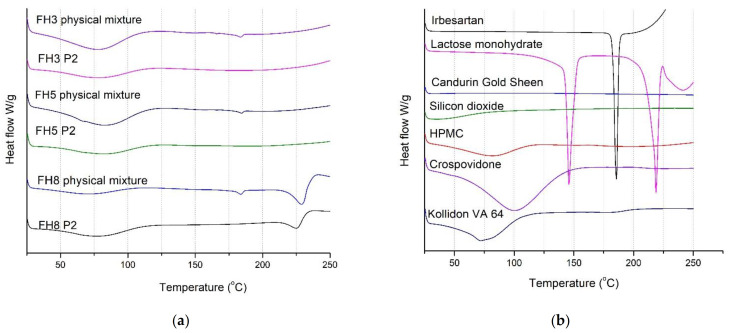
DSC curves of (**a**) selected physical mixtures and SLS irbesartan tablets, and (**b**) irbesartan and excipients.

In this study, DSC was used to verify the physical state of the drug in the formulations (Figure 8). The DSC curve of pure irbesartan exhibited single endothermic peaks at 185.67 °C, which is designated as the melting point of the irbesartan [44,45]. All evaluated formulations as well as the FH8 physical mixture showed broad endotherm corresponding to the water evaporation and polymer relaxation [12,46]. The FH8 physical mixture and FH8 P2 formulations containing lactose monohydrate showed an endothermic peak at 218–230 °C, which refers to the lactose melting [47]. In the FH3, FH5 and FH8 physical mixtures, a small endothermic peak at 181–186 °C is evident, suggesting the presence of irbesartan in crystalline form. No endothermic peak near 185 °C was detected in tablets prepared with the SLS 3D printing method (FH3 P2, FH5 P2 and FH8 P2), indicating that irbesartan is present in an amorphous form. The amorphization of drugs from their crystalline state can enhance the dissolution and bioavailability of low solubility drugs (BCS Class II or IV) since the amorphous state is generally associated with higher solubility [48,49]. A few studies reported the improvement of irbesartan’s solubility by converting it into an amorphous form as well as by preparing solid dispersions [50,51]. The amorphous form of irbesartan in the SLS tablets can be attributed to a conversion induced by an elevated temperature and laser irradiation during the printing process [8]. The amorphization of the drug in SLS printing has been reported in the literature. In a study by Fina et al., the conversion of paracetamol from a crystalline to amorphous state was observed in a mixture which also contained HPMC [12]. The presence of indomethacin in an amorphous form was observed in a study by Thakkar et al. on the granules obtained by a hot melt extrusion-based granulation process and SLS 3D printed tablets with these granules [16]. Moreover, the amorphous form of lopinavir in SLS printlets was detected in a study by Hamed et al. [9]. In this study, it was also shown that SLS could be used for the production of tablets with amorphous active substances.

#### 3.5.6. Disintegration Time

The time required for SLS tablets to disintegrate varied from 90.00 s to 1510.00 s (Table 3). The positive effect of ED on disintegration time was pronounced (Figure 4). With increasing laser speeds, a less energetic sintering process occurred during the printing process and particles in the tablets detached from each other more easily [12]. This is also reported in a study conducted by Mohamed et al. [7].

#### 3.5.7. Drug Content

The irbesartan content in all formulations was 85.00–115.00% of the theoretical content which indicates that there was no degradation of the drug during the sintering process (Table 3).

#### 3.5.8. Dissolution and Drug Release Kinetics

**Figure 9:**
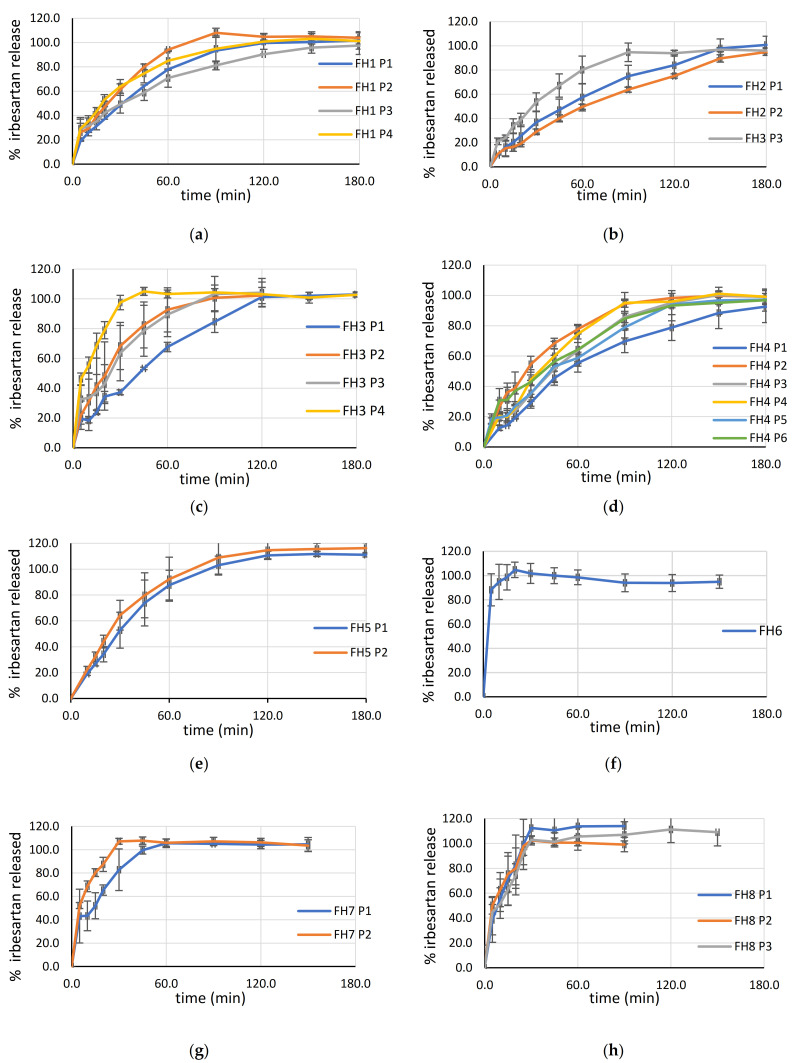
Drug release from irbesartan SLS tablets produced with different laser speeds: P1-100.00 mm/s, P2-120.00 mm/s, P3-140.00 mm/s, P4-160.00 mm/s, P5-180.00 mm/s, and P6-220.00 mm/s; (**a**) FH1, (**b**) FH2, (**c**) FH3, (**d**) FH4, (**e**) FH5, (**f**) FH6, (**g**) FH7, and (**h**) FH8.

The dissolution profiles of SLS tablets are provided in Figure 9. The correlation between disintegration time and drug release was observed. Tablets with a disintegration time of less than 325 s released irbesartan in less than one hour. It can be observed that FH8 P1, P2, and P3 reached complete release in less than 30 min. In a study conducted by Fina et al., formulations with HPMC produced with a laser speed of 300 mm/s exhibited the shortest dissolution profile, with complete drug release at approximately 2 h [12]. With SLS printing technologies and the adaption of formulation and process parameters, it was possible to produce tablets with HPMC as the main polymer, with complete drug release in less than 30 min.

#### 3.5.9. Effect of Crospovidone on Drug Release

**Figure 10:**
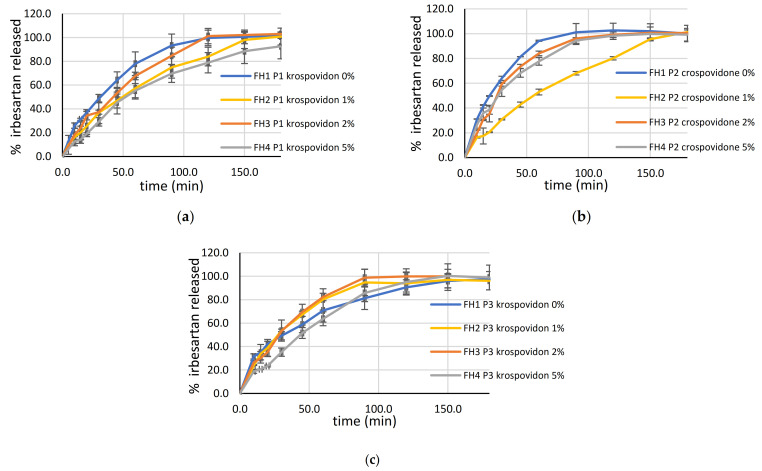
Effect of crospovidone on drug release: (**a**) tablets printed with a laser speed of 100.00 mm/s; (**b**) tablets printed with laser speed of 120.00 mm/s; (**c**) tablets printed with laser speed of 140.00 mm/s.

The developed decision tree showed the importance of crospovidone in printability. Surprisingly, crospovidone did not act as a super disintegrant in SLS printed tablets. The disintegration behavior of compacts containing crospovidone is attributed to the shape-memory characteristics of crospovidone [52]. Upon contact with water, crospovidone absorbs water via capillary action and regains its normal structure after releasing an amount of energy capable of breaking the tablet. The effect of crospovidone on SLS tablets was more complex. In Figure 10, drug release from tablets with the same laser speed and different concentrations of crospovidone is presented. It was observed that adding crospovidone leads to a decrease in drug release from SLS tablets. In tablets produced with a laser speed of 140.00 mm/s, drug release was slightly increased with the addition of crospovidone. The effect of crospovidone on disintegration and dissolution has been extensively investigated, mostly on tablets prepared with conventional technologies. Not only did formulation factors have an influence, the effects of crospovidone are also governed by the manufacturing process [53,54,55]. Compression pressure is reported as an important parameter for consideration [56,57]. To the best of our knowledge, the usage of crospovidone in SLS printing has not been reported previously in the literature. The melting point of crospovidone is close to the printing temperature; therefore, the SLS printing process probably enabled the melting of crospovidone and a better sintering process, which led to a decrease in the disintegration properties of crospovidone and, consequently, drug release.

#### 3.5.10. Tailoring Drug Release in SLS Printing

The effect of laser speed and, consequently, ED as a process parameter on drug release is shown in Figure 9. Increasing drug release with increasing laser speed and decreasing ED was pronounced in tablets FH2 and FH3, and was also noticeable in FH1 and FH4. In these tablets, which were printed at 140.00 mm/s, the drug is released significantly faster than tablets printed at 100.00 mm/s. Tuning the drug release by the parameters of the SLS process (surface temperature and laser scanning speed) has been performed for immediate release tablets [10]. Additionally, a similar observation was concluded in a study by [12]. However, FH5 (Kollidon VA 64 Fine 20%) was printable at 100.00 and 120.00 mm/s, and the effect of laser speed was not significant. In formulations containing HPMC and lactose monohydrate, SLS printing contributed to the production of voids through its medium and was able to penetrate fast, resulting in rapid drug release with a reduced impact of the laser speed. FH6 (lactose monohydrate 45%) was printable only with a laser speed of 100.00 mm/s. Complete release was reached in 30 min, but tablets had poor mechanical characteristics; thus, it was concluded that lactose monohydrate needed to be in a lower concentration. In tablets FH7 (lactose monohydrate 20%), a noticeable effect of laser speed on drug release was observed. However, laser speed did not have a significant influence on the drug release from FH8 P1, P2, and P3 tablets (lactose monohydrate 20% + crospovidone 2%); moreover, they reached complete release in less than 30 min. Due to the presence of crospovidone, printing was possible with a wider range of laser speeds. The effect of laser speed and ED was manifested on the physical characteristics of the tablets (previously discussed) rather than drug release. Hamed et al. observed that increasing the laser scanning speed from 75 to 100 mm/s, while keeping other variables constant, had no significant effect on the drug dissolution rate from tablets consisting of 50% drug, 35.5% Kolicoat IR, 10% lactose monohydrate, 1.5% talc and 3% Candurin^®^ NXT Ruby Red [9]. In conclusion, the tailoring of drug release in SLS printing might be achieved by formulation factors as well as process parameters, although it could be governed by the composition of the whole formulation.

#### 3.5.11. Drug Release Kinetics

To interpret the mechanism of drug release, data were fitted into various kinetic models such as zero-order, first-order, the Higuchi equation, and the Korsmeyer–Peppas equation (Table S1). The highest R^2^ coefficient determines the suitable mathematical model that best describes drug release kinetics and exponent **n** gave insights into the mechanism of drug release [58,59]. The most proper model fitted to data based on having the closest R^2^ to 1 was the Higuchi model for tablets from FH1, FH3, FH4 and FH5 formulations and Korsmeyer–Peppas for FH2, FH6, FH7 and FH8 formulations, but with very high values of R^2^ for the Higuchi model. Predominately, values for the exponent **n** were in the range from 0.45 to 0.89, indicating the combined drug release mechanisms of diffusion and erosion, or the so-called anomalous transport mechanism [60].

## 4. Conclusions

SLS is a challenging technique in terms of material selection and process parameters. It was observed that the printability of pharmaceutical powder mixtures was influenced by formulation characteristics and process conditions. The evaluation of energy density as critical process parameters in the SLS printing of solid dosage forms could allow the comparison of the results between different printers. This study demonstrated that decision tree methodology could be useful in understanding SLS printability. The most important factors that affected printability, according to the developed decision tree, were the content of crospovidone and ED. Tailoring tablets’ characteristics in SLS printing might be achieved by formulation factors as well as process parameters, although it needs to be governed by the composition of the whole formulation. The positive effect of ED on weight and disintegration time was observed; however, the impact of ED on drug release was reduced in formulations with lactose monohydrate. By adjusting the formulation and process parameters, it was possible to produce SLS tablets of macroporous structure with amorphous irbesartan and HPMC as the main polymer with complete drug release in less than 30 min. The obtained results indicate the potential of SLS printing in the production of tablets, and represent a closer step towards understanding and optimizing the SLS printing process.

## Figures and Tables

**Table 1:** Composition (% *w*/*w*) of the initial formulations.

	FM1	FM2	FH1	FH2	FH3	FH4	FH5	FH6	FH7	FH8
Irbesartan	5.0	5.0	5.0	5.0	5.0	5.0	5.0	5.0	5.0	5.0
Candurin Gold Sheen	3.0	3.0	3.0	3.0	3.0	3.0	3.0	3.0	3.0	3.0
Silicon dioxide	1.0	1.0	1.0	1.0	1.0	1.0	1.0	1.0	1.0	1.0
Mannitol	81.0	81.0	-	-	-	-	-	-	-	-
HPMC	-	-	91.0	90.0	89.0	86.0	71.0	46.0	71.0	69.0
Crospovidon	-	-	-	1.0	2.0	5.0	-	-	-	2.0
Kollidon VA 64 Fine	-	10.0	-	-	-	-	20.0	-	-	-
Lactose monohydrate	10.0	-	-	-	-	-	-	45.0	20.0	20.0

**Table 2:** SLS printing parameters for printing irbesartan SLS tablets.

Formulation	Printing Parameters				
	Chamber Temperature (°C)	Surface Temperature (°C)	Laser Speed (mm/s)	Layer Height (µm)	Energy Density (J/mm^3^)
FM1	150.00	160.00	60.00	200.00	0.77
FM2	125.00	140.00	50.00	200.00	0.92
FH1 P1	140.00	155.00	100.00	100.00	0.92
FH1 P2	140.00	155.00	120.00	100.00	0.77
FH1 P3	140.00	155.00	140.00	100.00	0.66
FH1 P4	140.00	155.00	160.00	100.00	0.57
FH2 P1	140.00	155.00	100.00	100.00	0.92
FH2 P2	140.00	155.00	120.00	100.00	0.77
FH2 P3	140.00	155.00	140.00	100.00	0.66
FH3 P1	140.00	155.00	100.00	100.00	0.92
FH3 P2	140.00	155.00	120.00	100.00	0.77
FH3 P3	140.00	155.00	140.00	100.00	0.66
FH3 P4	140.00	155.00	160.00	100.00	0.57
FH4 P1	140.00	155.00	100.00	100.00	0.92
FH4 P2	140.00	155.00	120.00	100.00	0.77
FH4 P3	140.00	155.00	140.00	100.00	0.66
FH4 P4	140.00	155.00	160.00	100.00	0.57
FH4 P5	140.00	155.00	180.00	100.00	0.51
FH4 P6	140.00	155.00	220.00	100.00	0.42
FH5 P1	115.00	120.00	100.00	100.00	0.92
FH5 P2	115.00	120.00	120.00	100.00	0.77
FH6	130.00	140.00	100.00	200.00	0.46
FH7 P1	140.00	155.00	100.00	100.00	0.92
FH7 P2	140.00	155.00	120.00	100.00	0.77
FH8 P1	140.00	155.00	100.00	100.00	0.92
FH8 P2	140.00	155.00	120.00	100.00	0.77
FH8 P3	140.00	155.00	140.00	100.00	0.66

**Table 3:** Physical and mechanical properties of irbesartan tablets.

Formulation	Weight ± SD (mg)	Diameter ± SD (mm)	Height ± SD (mm)	Disintegration Time (s)	Hardness ± SD (N)	Hausner Ratio	Compressibility Index (%)	Drug Content
**FM1**	n.d.	n.d.	n.d.	n.d.	n.d.	1.24	19.35	n.d.
**FM2**	n.d.	n.d.	n.d.	n.d.	n.d	1.14	12.5	n.d.
**FH1 P1**	35.1 ± 6.3	5.4 ± 0.1	2.6 ± 0.3	1510.0	56.0 ± 11.3	1.28	22.38	99.0 ± 0.9
**FH1 P2**	42.3 ± 8.1	5.15 ± 0.1	3.4 ± 0.3	1140.0	35.0 ± 31.1			100.5 ± 1.4
**FH1 P3**	44.7 ± 6.4	5.5 ± 0.1	3.4 ± 0.1	840.0	27.0 ± 10.5			101.9 ± 4.6
**FH1 P4**	44.0 ± 6.4	5.3 ± 0.3	3.5 ± 0.1	690.0	30.3 ± 11.1			99.6 ± 1.4
**FH2 P1**	32.2 ± 5.6	5.3 ± 0.2	2.8 ± 0.4	1000.0	51.0 ± 2.8	1.32	24.56	86.9 ± 0.7
**FH2 P2**	39.5 ± 8.3	5.2 ± 0.2	3.6 ± 0.4	720.0	48.3 ± 12.1			96.4 ± 1.0
**FH2 P3**	37.1 ± 8.5	5.3 ± 0.2	3.4 ± 0.3	460.0	n.d.			99.1 ± 2
**FH3 P1**	35.8 ± 4.1	5.5 ± 0.2	2.7 ± 0.4	660.0	45.3 ± 10.2	1.31	23.00	104.6 ± 0.5
**FH3 P2**	29.5 ± 6.5	5.4 ± 0.2	2.6 ± 0.2	500.0	n.d.			102.3 ± 3.1
**FH3 P3**	29.4 ± 6.7	5.4 ± 0.1	2.5 ± 0.3	380.0	n.d.			96.2 ± 2.3
**FH3 P4**	34.3 ± 4.4	5.3 ± 0.1	3.2 ± 0.2	420.0	n.d.			94.6 ± 3.2
**FH4 P1**	36.3 ± 6.5	5.4 ± 0.1	2.6 ± 0.5	1220.0	55.7 ± 15.3	1.31	23.7	89.2 ± 1.0
**FH4 P2**	26.7 ± 8.6	5.0 ± 0.2	2.0 ± 0.4	1040.0	27.7 ± 14.6			95.5 ± 3.3
**FH4 P3**	40.4 ± 9.1	5.3 ± 0.1	3.3 ± 0.3	960.0	51.0 ± 19.1			95.4 ± 2.9
**FH4 P4**	30.5 ± 7.2	5.3 ± 0.2	3.0 ± 0.3	600.0	n.d.			94.4 ± 0.1
**FH4 P5**	38.3 ± 5.7	5.1 ± 0.2	3.4 ± 0.2	540.0	n.d.			92.2 ± 0.9
**FH4 P6**	27.8 ± 10.2	5.0 ± 0.3	2.6 ± 0.5	480.0	n.d.			94.7 ± 0.5
**FH5 P1**	46.6 ± 12.3	5.4 ± 0.2	4.2 ± 0.4	1105.0	48.3 ± 14.1	1.29	22.49	107.2 ± 0.5
**FH5 P2**	43.8 ± 5.9	5.2 ± 0.5	4.2 ± 0.3	488.0	15.7 ± 2.1			113.0 ± 0.1
**FH6**	26.2 ± 7.1	5.3 ± 0.3	2.8 ± 0.2	240.0	n.d.	1.31	23.88	91.37 ± 0.5
**FH7 P1**	41.5 ± 6.7	5.5 ± 0.1	3.1 ± 0.0	780.0	n.d.	1.28	21.88	106.7 ± 6.5
**FH7 P2**	27.6 ± 7.2	5.0 ± 0.5	3.0 ± 0.6	220.0	n.d.			100.7 ± 2.9
**FH8 P1**	40.2 ± 7.2	5.6 ± 0.2	3.2 ± 0.3	260.0	12.5 ± 2.1	1.36	26.91	105.6 ± 7.9
**FH8 P2**	36.96 ± 6.3	5.5 ± 0.2	3.1 ± 0.2	180.0	n.d.			109.3 ± 0.6
**FH8 P3**	28.6 ± 3.9	5.4 ± 0.2	3.2 ± 0.2	90.0	n.d.			105.9 ± 5.2

n.d.—not determined.

## Data Availability

On request.

## Supplementary Materials

The following are available online at www.mdpi.com/article/10.3390/pharmaceutics13111969/s1, Table S1: Parameters obtained by fitting dissolution data to various mathematical models.
