# Supplementary material for: Understanding the Effect of Energy Density and Formulation Factors on the Printability and Characteristics of SLS Irbesartan Tablets—Application of the Decision Tree Model
Source: Pharmaceutics. 2021 Nov 20;13(11):1969. doi: 10.3390/pharmaceutics13111969 (PMC8621390; doi:10.3390/pharmaceutics13111969)
Supplement: Supplementary file 1 [file pharmaceutics-13-01969-s001.zip › pharmaceutics-1404377-supplementary.pdf]

pharmaceutics

Supplementary Material: Understanding the Effect of Energy Density and Formulation Factors on Printability and Characteristics of SLS Irbesartan Tablets—Application of Decision Tree Model

Marijana Madžarević, Đorđe Medarević, Stefan Pavlović, Branka Ivković, Jelena Đuriš and Svetlana Ibrić

Table S1. Parameters obtained by fitting dissolution data to various mathematical models.

Formulation	Zero order		First order		*Higuchi*		Korsmeyer – Peppas		
	k_0	r^2	k_1	r^2	K_h	r^2	k_{kp}	r^2	n
FH1 P1	0.4862	0.9343	0.0110	0.7980	7.8185	**0.9586**	4.5082	0.8400	0.5820
FH1 P2	1.0271	0.9535	0.0170	0.8667	12.3882	**0.9869**	9.5406	0.9763	0.5446
FH1 P3	0.4172	0.9182	0.0069	0.8367	6.8508	**0.9824**	13.6611	0.9800	0.3867
FH1 P4	0.6449	0.8921	0.0126	0.7949	9.7870	**0.9521**	5.3460	0.9260	0.6065
FH2 P1	0.4071	0.8869	0.0083	0.7312	7.6501	0.9695	3.9462	**0.9834**	0.6270
FH2 P2	0.4019	0.9302	0.0088	0.8003	7.4247	0.9832	3.4393	**0.9852**	0.6326
FH2 P3	0.4490	0.7960	0.0080	0.6939	7.6428	0.9151	9.4345	**0.9557**	0.4831
FH3 P1	0.7511	0.9786	0.0145	0.8742	10.7164	**0.9936**	3.8673	0.9898	0.6875
FH3 P2	0.8809	0.8845	0.0139	0.7972	11.6154	0.9574	9.1708	**0.9696**	0.5586
FH3 P3	0.9233	0.9356	0.0146	0.8657	11.9677	**0.9786**	8.6389	0.9752	0.5644
FH3 P4	1.0784	0.8293	0.0142	0.7802	11.5628	0.9192	25.7278	**0.9593**	0.3627
FH4 P1	0.5637	0.9471	0.0136	0.8166	8.9071	**0.9895**	1.9823	0.9798	0.7819
FH4 P2	0.4880	0.8656	0.0080	0.6631	8.1420	0.9471	9.5194	**0.9658**	0.4893
FH4 P3	0.6383	0.9432	0.0124	0.8477	10.0713	**0.9827**	3.3448	0.9792	0.6999
FH4 P4	0.6318	0.8774	0.0119	0.7690	10.1656	**0.9506**	3.7690	0.9487	0.6915
FH4 P5	0.5983	0.9528	0.0117	0.8560	9.4157	**0.9875**	3.9047	0.9850	0.6583
FH4 P6	0.5120	0.9444	0.0087	0.8870	8.0648	**0.9806**	9.1261	0.9792	0.4775
FH5 P1	0.8591	0.9041	0.0148	0.7786	12.6083	**0.9713**	3.8018	0.9710	0.7404
FH5 P2	0.8351	0.8821	0.0131	0.7436	12.3433	**0.9612**	5.6359	0.9559	0.6657
FH6	0.3432	0.4868	0.0038	0.4760	4.0126	0.6417	67.4411	**0.7938**	0.1126
FH7 P1	0.8743	0.9237	0.0180	0.9061	11.3225	**0.9645**	13.2711	0.9642	0.4722
FH7 P2	2.1190	0.9810	0.0272	0.9378	16.6109	0.9960	27.6331	0.9964	0.3934
FH8 P1	1.5629	0.9387	0.0278	0.9317	15.9325	0.9792	19.7032	**0.9823**	0.4678
FH8 P2	1.1162	0.8977	0.0197	0.8996	11.4342	0.9457	29.8048	**0.9660**	0.3344
FH8 P3	1.4679	0.9430	0.0283	0.9385	14.9307	0.9795	17.5138	**0.9866**	0.4756

k_0—zero order rate constant, k_1—first order rate constant, k_h—Higuchi dissolution constant, k_{kp}—Korsmeyer release rate constant, r^2—coeffcient of determination, n—drug release exponent.
